# Frequency and mutual associations of knee osteoarthritis features on ultrasound imaging in recent-onset symptomatic knee osteoarthritis patients: A cross-sectional analysis in a convenience sample of Dutch patients

**DOI:** 10.1016/j.ocarto.2025.100736

**Published:** 2025-12-22

**Authors:** Lianne Straetemans, Joost Verbruggen, Luc J.M. Heijnens, Jochen W.L. Cals, Chris L. de Korte, Thomas L.A. van den Heuvel, Ramon P.G. Ottenheijm

**Affiliations:** a Medical Ultrasound Imaging Center, Radboud University Medical Center, Nijmegen, the Netherlands; b Department of Family Medicine, Care and Public Health Research Institute, Maastricht University, Maastricht, the Netherlands; c Department of Orthopedic Surgery, Zuyderland Medical Centre, Sittard-Geleen, the Netherlands

**Keywords:** Ultrasonography, Osteoarthritis, Knee, General practice, Orthopedics, Diagnosis

## Abstract

**Objectives:**

To understand the diagnostic utility of ultrasound (US) for early diagnosis of knee osteoarthritis (KOA), this study aims to assess the frequency of KOA-associated US-detectable synovitis, osteophytes, and meniscal extrusion and their mutual association in patients diagnosed with recent-onset symptomatic KOA.

**Methods:**

The study included 476 patients (median age: 61) diagnosed with unilateral KOA in a recent-onset symptomatic (<2 years of pain) stage. All patients underwent an US exam assessing the presence of synovitis, osteophytes (lateral, medial, trochlear) and meniscus extrusion (medial, lateral), dichotomized as present or absent. A multivariate logistic regression analysis was performed to assess their mutual association.

**Results:**

The frequency of synovitis, osteophytes, and meniscal extrusion on US was 82.8 %, 87.0 %, and 44.7 %, respectively. At least one feature was detected in 97.7 % of the knees. All three features together were found in 32.6 % of the knees. In 73.1 % of the knees both synovitis and osteophytes were observed (odds ratio [OR] = 1.81, 95 % CI 0.95–3.45) and 37.8 % of the knees displayed meniscal extrusion along with synovitis (OR = 1.19, 95 % CI 0.73–1.94). Medial osteophytes alongside medial meniscal extrusion was identified in 30.5 % of the knees (OR = 1.26, 95 % CI 0.76–2.08), while lateral osteophytes alongside lateral extrusion was observed in 8.4 % of the knees (OR = 6.50, 95 % CI 3.31–12.77).

**Conclusions:**

Nearly all recent-onset symptomatic osteoarthritic knees exhibit at least one US-detectable KOA-associated feature. This underlines that even in KOA patients with short duration of pain, significant changes are already evident on US, offering potential support for earlier intervention and more targeted management strategies.

## 1 Introduction

Knee osteoarthritis (KOA) stands as one of the most prevalent musculoskeletal disorders, with a high burden of disease [1]. Despite its high prevalence, treatment options are currently mostly limited to lifestyle changes (e.g., weight reduction, diet changes, exercising), physical therapy, pain-relieving medication, promising procedures (e.g., radiofrequency ablation, genicular artery ablation) [2], intra-articular corticoid injections, or joint replacement in end-stage KOA [3]. Efforts to expand treatment options and to develop disease modifying drugs have not yet been successful, possibly due to the inclusion of patients based on radiographic criteria as often defined by Kellgren and Lawrence (KL) grade ≥2 [4,5]. At this stage, the general opinion is that tissue changes are irreversible and disease modifying drugs will not have the desired effect. This has resulted in a growing interest in early diagnosis of KOA, as it is believed that at this stage, disease progression can still be halted or possibly even reversed [6,7]. However, diagnosis of early-stage KOA has so far remained challenging, because yet, there is no clearly established definition of early-stage KOA available [7,8], and because of limitations of diagnostic methods.

Presently, diagnostic criteria and radiography are commonly used in clinics to diagnose KOA, but both methods are limited in identifying patients with early-stage KOA. Different sets of clinical diagnostic criteria exist, of which the best known are created by the American College of Rheumatology (ACR) [5], European League Against Rheumatism (EULAR) [9] and the National Institute for Health and Care Excellence (NICE) [10]. Differences exist between these sets of criteria and they show differences in performance to diagnose KOA [11,12]. Moreover, it is important to note that staging is not incorporated in these sets of diagnostic criteria. Additionally, previous research has demonstrated that the criteria cannot consistently diagnose KOA in a patient over time [11]. Radiographic evidence, such as reduced joint space and osteophyte formation, can supplement clinical assessment. However, radiography is unable to visualize soft tissue structures, including the synovium and meniscus, which are relevant structures for early KOA detection, since changes to these structures are already present in early stages of the disease [13,14].

Amidst this diagnostic conundrum, ultrasonography (US) emerges as a promising imaging tool, offering potential solutions to the diagnostic challenges. The role of US in the assessment of rheumatic and musculoskeletal disorders has increased as more and more rheumatologists use it as the first-choice imaging modality for diagnosing and monitoring [15,16]. US provides reliable assessment of KOA-associated features, as it is able to visualize detailed bony structures, such as small osteophytes, and soft tissue changes, including synovitis and meniscal extrusion. Studies have shown that US stands as a valid and reliable diagnostic modality for evaluating these KOA-associated features, with findings similar to those achieved through magnetic resonance imaging (MRI) [[17], [18], [19]].

The frequency of US detectable KOA features has been described in several studies. These studies report high frequencies of synovitis, osteophytes, and meniscal extrusion in patients that fulfilled ACR criteria or had radiographic KOA (KL grade of 2 or higher) [20,21], both of which are known to be indicative of late/end stage KOA. Nevertheless, data on US detectable features in early-stage KOA remains sparse [22].

In order to understand the diagnostic utility of US for diagnosis of early-stage symptomatic KOA, it is important to gain information on the frequency of KOA features in patients with recent-onset symptomatic KOA. Hence, the aim of this study was to assess the frequencies of US-detectable synovitis, osteophytes, and meniscal extrusion and their association in patients diagnosed with recent-onset symptomatic KOA. Within this work, recent-onset symptomatic KOA is defined by a pain duration of less than two years.

## 2 Methods

### 2.1 Study population

We conducted a cross-sectional study, retrospectively analyzing the medical records of adult patients diagnosed with unilateral KOA in a primary care orthopedic clinic in the Netherlands who were referred by their family physicians during the period January 2021 to June 2023. This clinic provides care for low-complexity cases for which the referring family physician does not expect a surgical indication. The diagnosis of KOA was based on clinical assessment by orthopedic surgeons familiar with knee treatment following the Dutch KOA guidelines [23], and a point-of-care US exam. The Dutch KOA guidelines are derived from the ACR, EULAR, and NICE clinical criteria for KOA [5,9,10]. Typical criteria for KOA included age 45 years or older, activity-related pain in the knee joint, and no or short-lasting (<30 min) morning stiffness related to knee joint. Additional features that increased the likelihood of a KOA diagnosis were pain and stiffness after inactivity (e.g., after sleeping or prolonged sitting). A clear and widely accepted definition of early KOA is currently lacking. Therefore, we defined “early” in our study as recent-onset symptomatic knee OA with a pain duration of less than two years, consistent with the approach used by Wang et al. [24] In addition, we followed the first phase of the OARSI initiative towards establishing criteria for early diagnosis by systematically excluding other potential diagnoses [25]. In atypical presentations, the diagnosis was made based on the presence of US features and the exclusion of other diagnoses. To diagnose KOA and consider other differential diagnoses, we only included patients who received a point-of-care US exam. All patients who met the predefined inclusion criteria within the specified time frame were included in the study. The exclusion criteria (bilateral knee pain, diagnoses other than KOA, absence of US examination, and pain duration exceeding two years) were applied consistently to ensure a homogeneous study population. This study was approved by the Medical Ethics Committee of Maastricht University (FHML-REC/2023/043); the requirement for patient consent was waived.

### 2.2 Data collection

The demographic characteristics, clinical data, and previous radiographic imaging findings by a radiologist were systematically collected from the GPs' referral letter and patient's medical record. The duration of pain was part of the anamnesis. Clinical data included history of inflammatory rheumatic disorder (e.g., rheumatoid arthritis, (pseudo)gout, and psoriatic arthritis), and history of metabolic disease (diabetes mellitus, obesity, hypertension, hypercholesterolemia). The results of the clinical diagnostic point-of-care US exams were collected retrospectively for this study.

### 2.3 US evaluation

**Fig. 1:**
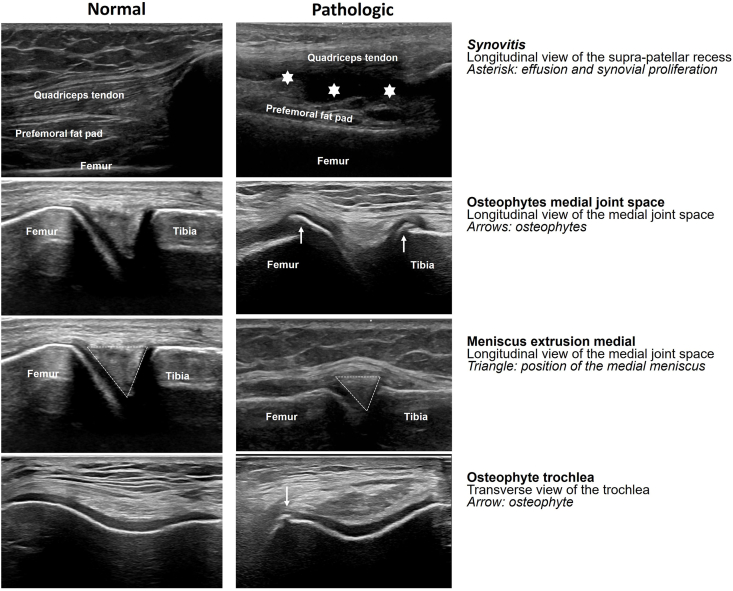
The appearance of the assessed KOA-associated features (synovitis, osteophytes and meniscus extrusion) on US images. On the left side, the normal tissue is shown; On the right side, the pathologies are imaged and indicated by asterisks (synovitis), arrows (osteophytes), and triangles (meniscus).

All point-of-care US exams of the knee joint were performed and analyzed as part of the clinical diagnostic process by one of two sonographers with 10 and 16 years’ experience in musculoskeletal US, respectively. The sonographers were not blinded to the clinical presentation. The US exams were performed using an Affiniti 70 US system (Philips Healthcare, Best, Netherlands) with a high-resolution, multi-frequency 4–18 MHz linear transducer. All US exams were based on a protocol derived from the Johnston county Osteoarthritis Project, the EULAR, and Outcome Measures in Rheumatology (OMERACT) guidelines for knee ultrasound [[26], [27], [28], [29]]. This is a fixed protocol with standardized probe positioning, with patients in supine position: longitudinal and transverse suprapatellar anterior in 30° flexion (subsequently scored for synovitis), medial and lateral joint space longitudinal in 30° flexion (for osteophytes and meniscal extrusion), and a maximally flexed suprapatellar transverse view (for trochlear osteophytes [30]). Synovitis was defined as increased hypoechoic or anechoic area that is displaceable and compressible in the suprapatellar recess at longitudinal scan. Although literature often distinguishes between effusion and synovial hypertrophy, we used a composite definition as both features reflect the same pathophysiologic process, because the composite definition showed a higher intra- and interobserver reliability [26], and because this is considered clinically more helpful to diagnose KOA. Osteophytes were defined as cortical protrusions close to the tibiofemoral joint space or in the trochlea. The meniscus was considered extruded when the outer edge extends beyond the femoral and tibial margin. Fig. 1 illustrates the ultrasound appearance of these features. Presence of these three features was dichotomized in either present or absent. For osteophytes and meniscal extrusion, the presence was also assessed per location. A dichotomous scale was used, since this was previously shown to have an excellent interobserver agreement [26,31], and to minimize the influence of clinical presentation on interpretation of the US images.

### 2.4 Outcome measures

The primary outcome of this study was the frequency of the following three US-detectable KOA-associated features: synovitis, osteophytes, and meniscal extrusion. For the latter two, both the overall frequency and location specific frequency were assessed. As a secondary outcome measure, the associations between different KOA-features were determined. Specifically, we assessed whether the presence of one feature was associated with the presence of another feature. This analysis was performed for the following pairs of features:

- Synovitis and location specific osteophytes,
- Synovitis and location specific meniscus extrusion,
- Trochlear osteophytes and meniscus extrusion,
- Medial osteophytes and medial meniscus extrusion, and
- Lateral osteophytes and lateral meniscus extrusion.

### 2.5 Statistical analysis

Continuous variables (i.e., age and pain duration) were assessed for normality using the Kolmogorov–Smirnov test and expressed as the mean and standard deviation or median and interquartile range, as appropriate. Additionally, to determine the association between the US-detectable KOA-associated features, odds ratios (OR) and the corresponding 95 % confidence intervals (CI) were calculated using a logistic regression with age, sex, duration of pain, history of inflammatory rheumatic disorder, and history of metabolic disease as covariates. All data were analyzed using IBM SPSS Statistics for Windows (IBM Corp, Armonk, NY, USA, version 29.0.0.0).

## 3 Results

### 3.1 Demographic and clinical characteristics

**Fig. 2:**
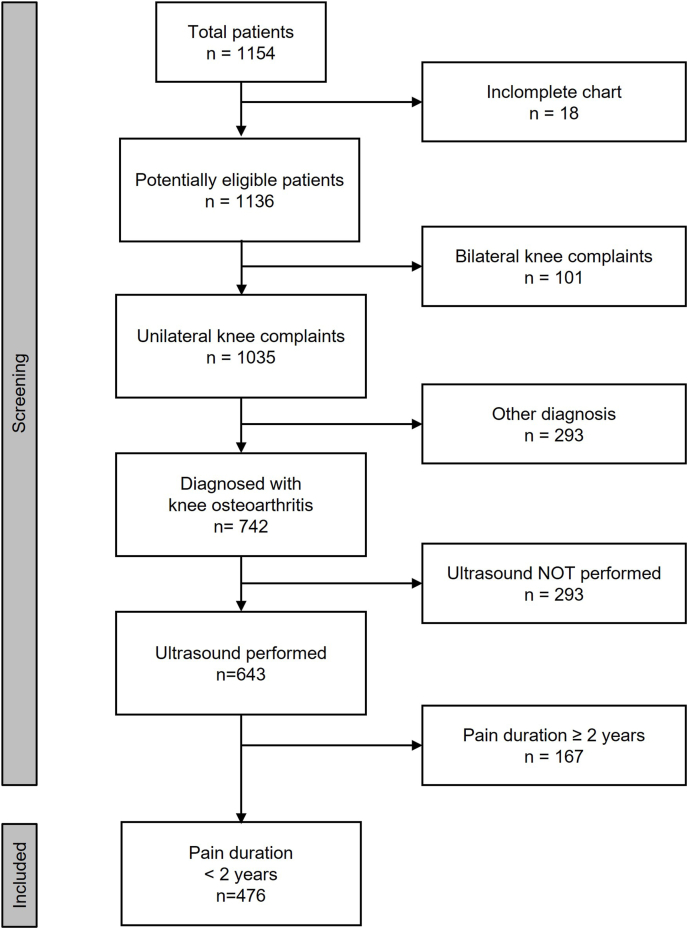
Flow diagram of the patient selection process.

The study population comprised 476 patients who were diagnosed with early-stage unilateral knee osteoarthritis. Fig. 2 shows a flow diagram of the patient selection process. Table 1 displays the baseline demographic, clinical, and imaging results ordered by the referring family physicians. The mean duration of pain was 12.0 weeks with an interquartile range of 20 weeks.

**Table 1:** Baseline characteristics (N = 476). IQR, interquartile range.

Characteristic	
Age (years), median (IQR)	61.0 (16.0)
Female sex, N (%)	272 (57.1)
Duration of complaints (weeks), median (IQR)	12.0 (20.0)
History of inflammatory rheumatic disorder, N (%)	29 (6.1)
History of metabolic disease, N (%)	221 (46.4)
Knee radiograph ordered by family physician, N (%)	63 (13.2)
Radiographic knee osteoarthritis present N (%)	39 (61.9)

### 3.2 Frequency of US-detectable KOA-associated features

**Fig. 3:**
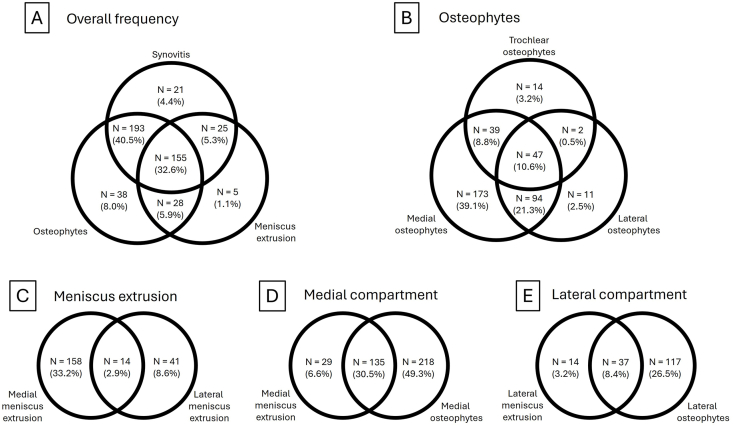
The frequency and overlap of US-detected synovitis, osteophytes, and meniscus extrusion. (A) shows the overall frequency and overlapping presence of the features, (B) shows the compartmental distribution of osteophytes, (C) shows the compartmental distribution of meniscus extrusion, (D)and (E) show the frequency and overlapping presence of meniscus extrusion and osteophytes in the medial compartment and lateral compartment, respectively. The patients with unspecified location of tibiofemoral osteophytes were excluded for (B), (D), and (E).

The frequencies of synovitis, osteophytes, and meniscal extrusion were 82.8 %, 87.0 %, and 44.7 %, respectively. The frequencies of the osteophyte location and side of meniscal extrusion are presented in Table 2. Fig. 3 presents a graphical overview of the frequencies and distributions of the US-detectable KOA-associated features.

**Table 2:** Prevalence of synovitis, osteophytes and meniscal extrusion, as well as the prevalence of osteophyte location and side of meniscal extrusion. For osteophytes, the table presents how many of the subgroup osteophytes were located at the tibiofemoral or trochlear joint. For the subgroup tibiofemoral joint osteophytes, the table presents the percentage of osteophytes that were located medial, lateral, or unspecified. For the subgroup meniscus extrusion, the table presents the percentage of meniscal extrusions observed medial and lateral.

	N (476)	% of total	% of subgroup
Synovitis	394	82.8 %	

Osteophytes	414	87.0 %	
tibiofemoral joint	400	84.0 %	96.6 %
medial	353	74.2 %	88.3 %
lateral	154	32.4 %	38.5 %
unspecified	34	7.1 %	8.5 %
trochlear	109	22.9 %	26.3 %

Meniscal extrusion	213	44.7 %	
medial	172	36.1 %	80.8 %
lateral	55	11.6 %	25.8 %

At least one of these three features was detected with US in 97.7 % of the knees. In the remaining 2.3 % of the knees, the diagnosis was made based on available radiographic imaging (N = 4) or other findings on US imaging (joint space narrowing (N = 1), irregular cartilage in the trochlea (N = 1) or degenerative meniscus (N = 5)). Synovitis, osteophytes, and meniscal extrusion were all found in 32.6 % of the knees. In addition, 81.9 % of the knees exhibited at least one feature of the medial compartment, 36.1 % displayed at least one feature of the lateral compartment, and 31.3 % of knees manifested at least one feature from both the medial and lateral compartment.

### 3.3 Association between US-detectable KOA-associated features

Our study showed that 73.1 % of the knees exhibited both synovitis and osteophytes, yielding an OR of 1.81 (95 % CI 0.95–3.45). Additionally, 37.8 % of the knees displayed meniscal extrusion along with synovitis, resulting in an OR of 1.19 (95 % CI 0.73–1.94). The occurrence of osteophytes of the medial compartment alongside medial meniscal extrusion was identified in 30.5 % of the knees with an OR of 1.26 (95 % CI 0.76–2.08), while osteophytes in the lateral compartment in conjunction with lateral extrusion were observed in 8.4 % of the knees exhibiting an OR of 6.50 (95 % CI 3.31–12.77). A complete overview of the associations is shown in Table 3.

**Table 3:** The association (odds ratio (OR)) between the different KOA-associated features. The statistically significant associations are shown in bold.

	Synovitis	Osteophytes	OsteophytesMedial joint space	OsteophytesLateral joint space	OsteophytesTrochlear
Osteophytes	1.81 (95 % CI 0.95–3.45)				
Osteophytes medial	**2.71 (95 % CI 1.56**–**4.72)**				
Osteophytes lateral	**2.01 (95 % CI 1.12**–**3.59)**				
Osteophytes trochlear	0.85 (95 % CI 0.49–1.49)				
Meniscus extrusion	1.19 (95 % CI 0.73–1.94)	0.79 (95 % CI 0.45–1.36)			0.72 (95 % CI 0.47–1.13)
Meniscus extrusionMedial	0.98 (95 % CI 0.59–1.62)		1.26 (95 % CI 0.76–2.08)		
Meniscus ExtrusionLateral	**2.89 (95 % CI 1.00**–**8.31)**			**6.50 (95 % CI 3.31**–**12.77)**	

## 4 Discussion

### 4.1 Frequency of KOA features

In this study, we evaluated the frequency and association of three KOA-associated features (synovitis, osteophytes, and meniscal extrusion) detected on US in a population of 476 patients diagnosed with symptomatic KOA and with a duration of pain of less than two years. Our findings show that nearly all (97.7 %) of symptomatic knees displayed at least one KOA feature on US, with osteophytes being the most observed (87.0 %), followed by synovitis (82.8 %), and meniscal extrusion (44.7 %). Additionally, osteophytes and meniscal extrusion were observed more in the medial compartment (74.2 % and 36.1 %) compared to the lateral compartment (32.4 % and 11.6 %), which is a trivial finding and corresponds with previous results presented in literature by Wang et al. [32].

Comparison with the only study reporting frequencies in patients with early KOA is somewhat challenging [22]. They report frequencies of 9.2 %, 38.8 %, 48.0 % and 61.2 % for synovial hyperplasia hypertrophy, knee joint effusion, and osteophytes of the medial condyle of femur and tibia, respectively. While in that study synovial hypertrophy and effusion are distinguished, we have combined these under the feature of synovitis and they have subdivided osteophytes of the medial joint space into femoral and tibial. However, it appears that we still find higher frequencies. A possible explanation may be the difference in study population: KOA patients with KL grade ≤1 versus KOA patients with a symptom duration of less than two years. One MRI study, by Hada et al. [33], including 50 patients with early-stage KOA reported a high frequency of osteophytes (98 %), which is in line with our findings. However, they included patients based on radiographic KL grade ≤2 whereas our study used the duration of symptoms to identify recent-onset symptomatic KOA patients. Since radiography data is only available for 13.2 % of our population, it is not possible to make a direct comparison between the studies mentioned. Oo et al. [20] examined the frequency of KOA features on US in patients with mild to moderate radiographic KOA with a mean disease duration of 8.9 years. They report a frequency of 95.5 %, 100 % and 100 % for synovitis, osteophytes and meniscal extrusion, respectively. A review by Sarmanova et al. [21] reports a pooled frequency of 58.7 % and 49.0 % for synovial effusion and synovial hypertrophy, respectively. Both studies, however, only included patients that fulfilled ACR criteria or had radiographic KOA, both of which are known to be indicative of a late stage of the disease [7,34]. Therefore, their study population cannot be compared directly to our recent-onset symptomatic KOA study population.

### 4.2 Synovitis vs. meniscal extrusion

Between lateral meniscus extrusion and synovitis, a statistically significant positive association was found (OR = 2.89, 95 % CI 1.00–8.31), while a non-significant negative association was found between medial meniscal extrusion and synovitis (OR = 0.98, 95 % CI 0.59–1.62). Interestingly, a study by Grainger et al. [35] has shown an association between synovitis and meniscal extrusion in the medial compartment of the joint, but a less strong association between meniscal extrusion and synovitis in the lateral compartment. Additionally, research by Deng et al. [36], in which 589 patients with various radiographic KOA grades were studied, suggests that medial meniscus extrusion affects synovitis. Although our results show a stronger association in the lateral compartment of the knee joint than in the medial compartment, our results add to existing evidence that meniscal extrusion and synovitis are interacting.

### 4.3 Synovitis vs. osteophytes

For synovitis and osteophytes, a statistically significant positive association was found between synovitis and osteophytes in the medial compartment (OR = 2.71, 95 % CI 1.56–4.72), as well as between synovitis and osteophytes in the lateral compartment of the knee joint (OR = 2.01, 95 % CI 1.12–3.59). This corresponds with previous research by Kaneko et al. who found that synovitis serves as a risk factor for the formation and development of osteophytes [37].

### 4.4 Meniscal extrusion vs osteophytes

A statistically significant positive association was found between lateral meniscal extrusion and lateral osteophytes (OR = 6.50, 95 % CI 3.31–12.77), while a non-significant positive association was found between medial meniscal extrusion and medial osteophytes (OR = 1.26, 95 % CI 0.76–2.08). This in contrary to previous studies that found a positive association between medial meniscal extrusion and osteophytes in the medial compartment [33, 38]. Hada et al. [33] reports a positive association between medial osteophytes and medial meniscal extrusion in patients with early-stage KOA. However, as described earlier, this study identified early-stage KOA patients by radiographic grading. Hence, their study population cannot be compared directly to the population in this study and differences in statistical significance might be explained by differences in stage of KOA included.

### 4.5 Clinical implications

The clinical implication of our findings is that US is highly effective in detecting key features of KOA in symptomatic patients with a relatively short pain duration, including in a setting where patients’ knees had not been previously diagnosed with OA, with nearly all affected knees showing at least one characteristic on US. This underscores the potential role of US as a valuable, non-invasive diagnostic tool for KOA, particularly in cases where conventional radiography is not available at the point-of-care, like primary care, or is inconclusive when early changes need to be identified. Especially when radiographs show little or no deformity (KL grade 0 or 1), structural changes in cartilage, subchondral bone, and meniscus may already be present. Integrating US in the diagnostic pathway of patients with knee pain may support earlier intervention and more targeted management strategies for symptomatic KOA patients. In recent years, early intervention has gained significant scientific attention. Knowledge of the frequency of KOA-associated features on US in patients with short duration of symptoms is essential for guiding research efforts aimed at developing new diagnostic and therapeutic approaches. Especially understanding the frequency in recent-onset symptomatic KOA patients could be valuable for future research on intervention and disease-modifying therapeutic strategies [39].

Although US provides a valuable imaging tool in diagnosing KOA, performing an US, and interpreting the correct US images to identify KOA requires training and sonographer experience. Moreover, it is time-consuming, with reported scanning and interpretation taking approximately 10 min [40]. Artificial intelligence (AI) might help to minimize sonography training and make it a more accessible tool in primary care. Understanding the frequency of US-detectable KOA features helps future research on AI focus on the relevant KOA-features.

### 4.6 Strengths and limitations

The main study strengths are the relatively large population of 476 patients included in this study and the inclusion of KOA patients based on short duration of pain. Although it is debatable whether a pain duration of less than two years reflects early-stage KOA, a key strength of our study is the relatively short pain duration in our population. With a median duration of 12 weeks and an interquartile range of 20 weeks, 75 % of the population reported pain for less than 22 weeks, supporting the inclusion of patients at a relatively early disease stage. Only the study by Deng et al., and by D'Agostino et al. were found to study a larger population. Deng et al. evaluated 589 patients with various KL grades, mainly ranging from 1 to 3. The study by D'Agostino et al. included 600 patients. This study, however, describes a mean disease duration of 6.0 years and a KL grade ≥3 for 399/600 patients. By including patients with a short duration of pain, our study adds valuable information to this previous research and provides important information to the body of knowledge on diagnosing KOA as early as possible after first presentation of pain. Another strength was the use of a standardized scanning protocol with a dichotomous scale for assessing the presence of synovitis, osteophytes and meniscal extrusion, since this was shown to have an excellent interobserver agreement [41].

Several limitations of the present study should be noted. As cartilage degradation is often considered a key characteristic of KOA, it would possibly have been of value to include cartilage thickness measurements. However, this was not part of the US evaluation, because US-based cartilage thickness measurement is difficult to standardize and needs further research [26]. Moreover, meniscal extrusion was only examined in supine position and not in weight-bearing (standing) position. Medial meniscal extrusion is shown to increase in loaded knees, especially in those with minimal osteoarthritis [42]. Hence, loaded imaging may help to more accurately determine minimal extrusion and therefore, weighted imaging might be useful for diagnosis of early-stage KOA. In our study, this might have led to an underestimation of the frequency of meniscal extrusion. Another limitation regarding meniscal extrusion is the lack of quantitative measurement. Extrusion was assessed qualitatively (present/absent) rather than by applying a specific cut-off value. Although this approach aligns with pragmatic ultrasound practice, it may have led to an overestimation of the frequency of meniscal extrusion in patients with KOA, as minor degrees of extrusion can occur physiologically. It is recommended to include this in future research. Another limitation is that data collection was performed retrospectively rather than prospectively, potentially affecting the homogeneity of the data quality and thereby increasing the risk of missing information. However, a standardized scanning protocol was used by both experienced sonographers, hence the risk of having missed essential information is limited. Moreover, the data was collected in a single clinical setting, resulting in limited generalizability of the results outside this setting. Furthermore, the sonographers were not blinded for the clinical presentation, and available radiographs in the minority of patients, when performing the US assessment. This might have affected the interpretation of the US images by the sonographer. However, this is a key part of the diagnostic process as in this clinic the sonographer supports the integration of the clinical presentation with US findings to create a comprehensive clinical picture. Additionally, by using a dichotomous scale rather than a gradual scale, this influence was minimized. The final limitation of this study lies in the potential for diagnostic circularity, as the US, which was used to identify the KOA features, also played a role in confirming the diagnosis in several patients. This dual use of the US could introduce bias, with the results potentially influencing the diagnosis and thereby overestimating the frequency of US-detectable KOA features. For a future study, it would be valuable to include a larger population of KOA patients with short duration of symptoms for whom both radiographic images and US data is available, as this would also allow for a comparison between US and radiography. Moreover, that would enable the use of a radiography based definition of early osteoarthritis, e.g. KL < 2. The latter was not feasible in the present study, as radiographic imaging was not routinely performed in the setting where this study was conducted.

### 4.7 Conclusion

This study provides valuable insight into the frequency of US-detectable KOA features (synovitis, osteophytes, and meniscal extrusion) in a large group of patients with recent-onset symptomatic KOA. The findings reveal that nearly all symptomatic knees exhibited at least one of these features. Consequently, these results underscore that even in KOA patients with short duration of symptoms, significant changes are already evident, offering potential support for earlier intervention and more targeted management strategies.

## Authors contribution

All authors included in this study met the authorship criteria according to the ICJME recommendations (https://www.icmje.org).

Ramon P.G. Ottenheijm, Joost Verbruggen, Lianne Straetemans, Jochen W.L. Cals, Chris L. de Korte and Thomas L.A. van den Heuvel were responsible for the conception, design and methodology of this study.

Ramon P.G. Ottenheijm and Joost Verbruggen were responsible for collection and curation of data.

Lianne Straetemans was primarily responsible for data analysis and Ramon P.G. Ottenheijm provided feedback regarding data analysis and interpretation.

All authors were involved in data interpretation, and reviewed, edited, and approved the final manuscript.

## Role of funding source

This research is supported by ReumaNederland and NWO, as part of their joint strategic research program: KIC Early Detection of Osteoarthritis: Innovations for Detection and Diagnosis. Grant number: KICH2.V4P.RNL22.006. ReumaNederland and NWO did not take part in the study design, collection, analysis, and interpretation of data, writing of the report or the decision to submit the article for publication.

## Conflict of interest statement

Thomas L.A. van den Heuvel is founder, CEO and shareholder of Ardim B.V.. The other authors have no conflicts of interest to declare.

**Key messages**

*What is already known on this topic*

Diagnostic criteria and radiography are limited in identifying patients with early-stage KOA. Ultrasonography (US) provides reliable assessment of knee osteoarthritis (KOA) associated features, but data on US detectable features in recent-onset symptomatic KOA patients remains sparse.

*What this study adds*

Based on ultrasound exams of 476 patients with recent-onset symptomatic KOA, we have shown that nearly all symptomatic knees exhibit at least one ultrasound-detectable KOA-associated feature.

*How this study might affect research, practice or policy*

These findings support the use of US in the diagnostic workflow for patients with knee pain, aiding in the early detection of symptomatic KOA and enabling earlier, more targeted interventions.

## References

[1] Disease G.B.D., Injury I., Prevalence C. (2016). Global, regional, and national incidence, prevalence, and years lived with disability for 310 diseases and injuries, 1990-2015: a systematic analysis for the global burden of disease study 2015. Lancet.

[2] Kwak D.H., Hofmann H.L., Patel M., Heller D.B., Lyons A., Yu Q. (2024). Genicular artery embolization, radiofrequency ablation, and corticosteroid therapy for knee osteoarthritis: a cost-effectiveness analysis using randomized clinical trial data. AJR Am. J. Roentgenol..

[3] Gelber A.C. (2024). Knee osteoarthritis. Ann. Intern. Med..

[4] Oo W.M., Yu S.P., Daniel M.S., Hunter D.J. (2018). Disease-modifying drugs in osteoarthritis: current understanding and future therapeutics. Expet Opin. Emerg. Drugs.

[5] Altman R., Asch E., Bloch D., Bole G., Borenstein D., Brandt K. (1986). Development of criteria for the classification and reporting of osteoarthritis. Classification of osteoarthritis of the knee. Diagnostic and therapeutic criteria committee of the American rheumatism association. Arthritis Rheum..

[6] Oei E.H.G., Runhaar J. (2023). Imaging of early-stage osteoarthritis: the needs and challenges for diagnosis and classification. Skelet. Radiol..

[7] Mahmoudian A., Lohmander L.S., Mobasheri A., Englund M., Luyten F.P. (2021). Early-stage symptomatic osteoarthritis of the knee - time for action. Nat. Rev. Rheumatol..

[8] Mahmoudian A., King L.K., Liew J.W., Wang Q., Appleton C.T., Englund M. (2024). Timing is everything: towards classification criteria for early-stage symptomatic knee osteoarthritis. Osteoarthr. Cartil..

[9] Zhang W., Doherty M., Peat G., Bierma-Zeinstra M.A., Arden N.K., Bresnihan B. (2010). EULAR evidence-based recommendations for the diagnosis of knee osteoarthritis. Ann. Rheum. Dis..

[10] (2014). Osteoarthritis: Care and Management in Adults London.

[11] Wang Q., Runhaar J., Kloppenburg M., Boers M., Bijlsma J.W.J., Bierma-Zeinstra S.M.A. (2024). Evaluation of the diagnostic performance of American college of rheumatology, EULAR, and national institute for health and clinical excellence criteria against clinically relevant knee osteoarthritis: data from the CHECK cohort. Arthritis Care Res..

[12] Skou S.T., Koes B.W., Gronne D.T., Young J., Roos E.M. (2020). Comparison of three sets of clinical classification criteria for knee osteoarthritis: a cross-sectional study of 13,459 patients treated in primary care. Osteoarthr. Cartil..

[13] Englund M., Roemer F.W., Hayashi D., Crema M.D., Guermazi A. (2012). Meniscus pathology, osteoarthritis and the treatment controversy. Nat. Rev. Rheumatol..

[14] Mathiessen A., Conaghan P.G. (2017). Synovitis in osteoarthritis: current understanding with therapeutic implications. Arthritis Res. Ther..

[15] Oo W.M., Bo M.T. (2016). Role of ultrasonography in knee osteoarthritis. J. Clin. Rheumatol..

[16] McAlindon T., Kissin E., Nazarian L., Ranganath V., Prakash S., Taylor M. (2012). American college of rheumatology report on reasonable use of musculoskeletal ultrasonography in rheumatology clinical practice. Arthritis Care Res..

[17] Oo W.M., Linklater J.M., Bennell K.L., Daniel M.S., Pryke D., Wang X. (2022). Reliability and convergent construct validity of quantitative ultrasound for synovitis, meniscal extrusion, and osteophyte in knee osteoarthritis with MRI. J. Ultrasound Med..

[18] Keen H.I., Wakefield R.J., Conaghan P.G. (2009). A systematic review of ultrasonography in osteoarthritis. Ann. Rheum. Dis..

[19] Iagnocco A., Perricone C., Scirocco C., Ceccarelli F., Modesti M., Gattamelata A. (2012). The interobserver reliability of ultrasound in knee osteoarthritis. Rheumatology.

[20] Oo W.M., Linklater J.M., Bennell K.L., Pryke D., Yu S., Fu K. (2021). Are OMERACT knee osteoarthritis ultrasound scores associated with pain severity, other symptoms, and radiographic and magnetic resonance imaging findings?. J. Rheumatol..

[21] Sarmanova A., Hall M., Moses J., Doherty M., Zhang W. (2016). Synovial changes detected by ultrasound in people with knee osteoarthritis - a meta-analysis of observational studies. Osteoarthr. Cartil..

[22] Mizuno Y., Takata Y., Shima Y., Goshima K., Kuroda K., Kanayama T. (2025). Relationship between ultrasonographic findings and subscales of the knee injury and osteoarthritis outcome score in patients with early knee osteoarthritis: a multicenter study. J. Med. Ultrason..

[23] Specialisten F.M. (2018). Conservatieve behandeling van artrose in heup of knie. Feder. Med. Special..

[24] Wang Q., Runhaar J., Kloppenburg M., Boers M., Bijlsma J.W.J., Bierma-Zeinstra S.M.A. (2021). Diagnosis of early stage knee osteoarthritis based on early clinical course: data from the CHECK cohort. Arthritis Res. Ther..

[25] Hawker G.A., King L.K., Liew J.W., Wang Q., Mahmoudian A., Jansen N.E.J. (2025). OARSI initiative to develop classification criteria for early-stage symptomatic knee OA (EsSKOA): what conditions should be considered in the differential diagnosis of EsSKOA?. Osteoarthr. Cartil..

[26] Bruyn G.A., Naredo E., Damjanov N., Bachta A., Baudoin P., Hammer H.B. (2016). An OMERACT reliability exercise of inflammatory and structural abnormalities in patients with knee osteoarthritis using ultrasound assessment. Ann. Rheum. Dis..

[27] D'Agostino M.A., Conaghan P., Le Bars M., Baron G., Grassi W., Martin-Mola E. (2005). EULAR report on the use of ultrasonography in painful knee osteoarthritis. Part 1: prevalence of inflammation in osteoarthritis. Ann. Rheum. Dis..

[28] Nevalainen M.T., Uusimaa A.P., Saarakkala S. (2023). The ultrasound assessment of osteoarthritis: the current status. Skelet. Radiol..

[29] Yerich N.V., Alvarez C., Schwartz T.A., Savage-Guin S., Renner J.B., Bakewell C.J. (2020). A standardized, pragmatic approach to knee ultrasound for clinical research in osteoarthritis: the Johnston county osteoarthritis project. ACR Open Rheumatol..

[30] Kazam J.K., Nazarian L.N., Miller T.T., Sofka C.M., Parker L., Adler R.S. (2011). Sonographic evaluation of femoral trochlear cartilage in patients with knee pain. J. Ultrasound Med..

[31] Iagnocco A., Perricone C., Scirocco C., Ceccarelli F., Modesti M., Gattamelata A. (2012). The interobserver reliability of ultrasound in knee osteoarthritis. Rheumatology.

[32] Wang B., Liu Q., Wise B.L., Ke Y., Xing D., Xu Y. (2018). Valgus malalignment and prevalence of lateral compartmental radiographic knee osteoarthritis (OA): the Wuchuan OA study. Int. J. Rheum. Dis..

[33] Hada S., Ishijima M., Kaneko H., Kinoshita M., Liu L., Sadatsuki R. (2017). Association of medial meniscal extrusion with medial tibial osteophyte distance detected by T2 mapping MRI in patients with early-stage knee osteoarthritis. Arthritis Res. Ther..

[34] Peat G., Thomas E., Duncan R., Wood L., Hay E., Croft P. (2006). Clinical classification criteria for knee osteoarthritis: performance in the general population and primary care. Ann. Rheum. Dis..

[35] Grainger A.J., Rhodes L.A., Keenan A.M., Emery P., Conaghan P.G. (2007). Quantifying peri-meniscal synovitis and its relationship to meniscal pathology in osteoarthritis of the knee. Eur. Radiol..

[36] Deng H., Chen Z., Kang J., Liu J., Chen S., Li M. (2024). The mediating role of synovitis in meniscus pathology and knee osteoarthritis radiographic progression. Sci. Rep..

[37] Kaneko H., Adili A., Aoki T., Liu L., Negishi Y., Tomura J. (2022). osteophyte formation progresses when synovitis was stronger in middle-aged populations with grade 0 of kellgren-lawrence classification -the osteoarthritis initiative. Osteoarthr. Cartil..

[38] Negishi Y., Kaneko H., Aoki T., Liu L., Adili A., Arita H. (2023). Medial meniscus extrusion is invariably observed and consistent with tibial osteophyte width in elderly populations: the Bunkyo health study. Sci. Rep..

[39] Cho Y., Jeong S., Kim H., Kang D., Lee J., Kang S.-B. (2021). Disease-modifying therapeutic strategies in osteoarthritis: current status and future directions. Exp. Mol. Med..

[40] Menyawi M., Gamal G., Abdelbadie H., Elgohary R. (2024). Assessment of validity, reliability, and feasibility of OMERACT ultrasound knee osteoarthritis scores in Egyptian patients with primary knee osteoarthritis. Clin. Rheumatol..

[41] Oo W.M., Linklater J.M., Hunter D.J. (2017). Imaging in knee osteoarthritis. Curr. Opin. Rheumatol..

[42] Patel R., Eltgroth M., Souza R.B., Zhang C.A., Majumdar S., Link T.M. (2016). Loaded versus unloaded magnetic resonance imaging (MRI) of the knee: effect on meniscus extrusion in healthy volunteers and patients with osteoarthritis. Eur. J. Radiol. Open.

